# Editorial: Modern Lifestyle and Health: How Changes in the Environment Impacts Immune Function and Physiology

**DOI:** 10.3389/fimmu.2021.762166

**Published:** 2021-10-26

**Authors:** Laurence Macia, Olivier Galy, Ralph Kay Heinrich Nanan

**Affiliations:** ^1^ Faculty of Medicine and Health, The University of Sydney, Darlington, NSW, Australia; ^2^ Charles Perkins Centre, The University of Sydney, Camperdown, NSW, Australia; ^3^ School of Education, The University of New Caledonia, Nouméa, France

**Keywords:** environment, health, immunity, nutrition, physical activity

Physical fitness as well as nutritional access are major evolutionary drivers across all animal species. In modern societies both of these factors have become highly modifiable principally allowing survival despite a sedentary lifestyle in an environment of virtually limitless caloric access. This has led to an evolutionary mismatch for which we are now paying the price of increasing non-communicable diseases. In addition, we are also paying the price in terms of the enormous carbon footprints transport emmissions and industrialised animal agriculture are causing. In the Research Topic *Modern Lifestyle and Health: How Changes in the Environment Impacts Immune Function and Physiology* several studies have been published providing further evidence on how this discordance between our evolutionray programming and the adoption of a modern lifestyle influence our health and wellbeing.

A series of observational investigations focused on comparing more traditional rural to urban settings in developing countries in Pacific Islands Countries and Territories. The study by Bang Nguyen Pham et al. analysed diets in under 5-year-old children in Paupua New Guinea. Interestingly dietary diversity as a proxy measure of dietary adequacy was higher in the rural settings *versus* more westernized urban settings. Dietary diversity of urban children markedly increased with maternal educational status and wealth. This was then independently reflected in childhood growth patterns, with wasting and stunting being more prevalent in urban settings and compensated again by household socioeconomic status in the urban environment (Bang Nguyen Pham et al.). Also, in terms of physical activity Wattelez et al. in a study on adolescents in New Caledonia showed that living in rural areas was associated with more physical activities and less sitting time, in contrast to urban environments associated with a more sedentary lifestyle. The common theme here is obviously that negative impacts of low physical activity and nutrition of a modern lifestyle can be partially counteracted by education and socioeconomic factors.

Physical activity partially reverse conditions associated with our modern lifestyle and can be employed as preventive strategies or medical interventions. The study by Jabbour, suggests that vigorous physical activity in type 1 diabetic adolescents did not only improve long-term blood sugar levels but also had a favourable psychological impact on fear of hypoglycaemia. In addition, Sooyeon Oh et al. showed that physical activity and vitamin D were associated with an increase in natural killer cell activity. These cells are targeting cells under stress such as tumor cells or virus infected cells, hence, supporting the already known benefits of outdoor physical activity often lacking in modern societies. Furthermore, Leandro dos Santos et al. demonstrated that modified forms of resistant exercise, in which blood flow to the trained muscle groups is restricted, result in an increase in lymphocyte mobilisation in the circulation. The lymphocytes are then readily available for tissue reparation, opening the door to new rehabilitation strategies by harnessing the power of immune reparation. Physical activity has indeed systemic impacts. Ma et al., reviewed the effects of the myokin irisin on cardiovascular health and its potential use as a diagnostic in cardiovascular diseases.

León, et al. provided evidence that adaptive processes towards modern diets are beginning to evolve. They investigated how wheat, a staple nutritional component of modern societies, affects cell homeostasis through the splicing of pre-mRNAs encoding key regulatory proteins.

A further series of publications were focused on the effects of diet on the immune system and more broadly on immunometabolism. In contrast to traditional plant based diets, processed foods in westernized societies are typically low in dietary fibre. However, dietary fibre influences the gut microbiome composition and is the main source of energy for intestinal bacteria. Dietary fibre is typically fermented by gut bacteria resulting in the production of short chain fatty acids (SCFA), known to have profound immunomodulatory effects beneficial in diseases such as allergies, autoimmune diseases and cancer (1). Malczewski et al. specifically focused their review on dietary fibre in the context of cancers and discuss the link between response to cancer treatments and specific gut microbiota signature. They propose that the gut microbiota metabolome signature, particularly the profile of short chain fatty acids could be a better tool to identify therapeutic responders from non-responders.

Impact of fatty acids on immunometabolism was further investigated by Xu et al, and Sawada et al. looking at the role of unsaturated long chain fatty acids on the immune system. Macrophages are highly responsive to their environment and adopt either a M1 proinflammatory profile characterised by a glycolytic activity or a M2 anti-inflammatory profile characterised by oxidative phosphorylation and fatty acid biosynthesis (2). Xu et al. identified that arachidonic acid metabolism was enriched in M2 macrophages and that manipulation of this pathway can promote or inhibit M2 differentiation. Interestingly, solid tumours can have immunomodulatory effects through these mechanisms. Furthermore, Sawada et al. reviewed the anti-inflammatory role of fatty acids, in particular Omega_3 fatty acids on different immune cell subsets. They provided an overview on how Omega_3 fatty acids and resolvins, products of Omega_3 fatty acids metabolism, affect immune cell survival, activity and migration as well as their therapeutic potentials in skin conditions including psoriasis, dermatitis, wound healing.

Finally, identifying strategies to prevent disease development associated with industrialised agricultural livestocks is critical. In this context Lagos et al. proposed feeding weaning pigs on a yeast protein diet could have numerous benefits. On top of being an environmentally friendly source of protein, it also improved gut homeostasis in weaning piglets with little to no impact on their immunity. Weaning, piglets are at high risks of developing inflammatory diseases requiring heavy use of antibiotics, which then enters the human food chain and is associated risks of antibiotic resistance. Hence there approach might alleviate the environmental impact of meat production as well as have direct health effects on humans.

To conclude, this Research Topic highlighted that further research is required to elucidate the effects of modern lifestyle on health and wellbeing of us humans but beyond on the survival of the planet. When contrasting our evolutionary past to the modern lifestyle of sedentary behaviour and nutritional abundance it becomes clear that there are more lessons to be learnt to address the health challenges of our time.

## Author Contributions

LM, OG, RN were editors for this topic and wrote the commentary. All authors contributed to the article and approved the submitted version.

## Funding

Grant ARC LP160100627 and ARC DP210102943.

## Conflict of Interest

The authors declare that the research was conducted in the absence of any commercial or financial relationships that could be construed as a potential conflict of interest.

## Publisher’s Note

All claims expressed in this article are solely those of the authors and do not necessarily represent those of their affiliated organizations, or those of the publisher, the editors and the reviewers. Any product that may be evaluated in this article, or claim that may be made by its manufacturer, is not guaranteed or endorsed by the publisher.
